# Magnetic orientation of the Common Toad: establishing an arena approach for adult anurans

**DOI:** 10.1186/1742-9994-8-6

**Published:** 2011-03-21

**Authors:** Lukas Landler, Günter Gollmann

**Affiliations:** 1 University of Vienna, Department of Evolutionary Biology, 1090 Vienna, Althanstraße 14 (UZA I), Austria

## Abstract

**Background:**

Magnetic orientation is a taxonomically widespread phenomenon in the animal kingdom, but has been little studied in anuran amphibians. We collected Common Toads (*Bufo bufo*) during their migration towards their spawning pond and tested them shortly after displacement for possible magnetic orientation in arena experiments. Animals were tested in two different set-ups, in the geomagnetic field and in a reversed magnetic field. To the best of our knowledge, this is the first study testing orientation of adult anurans with a controlled magnetic field of a known strength and alignment.

**Results:**

After displacement, toads oriented themselves unimodally under the geomagnetic field, following their former migration direction (d-axis). When the magnetic field was reversed, the distribution of bearings changed from a unimodal to a bimodal pattern, but still along the d-axis. The clustering of bearings was only significant after the toads reached the outer circle, 60.5 cm from their starting point. At a virtual inner circle (diameter 39 cm) and at the start of the experiment, orientation of toads did not show any significant pattern.

**Conclusions:**

The experimental set-up used in our study is suitable to test orientation behaviour of the Common Toad. We speculate that toads had not enough time to relocate their position on an internal map. Hence, they followed their former migration direction. Bimodality in orientation when exposed to the reversed magnetic field could be the result of a cue conflict, between magnetic and possibly celestial cues. For maintaining their migration direction toads use, at least partly, the geomagnetic field as a reference system.

## Background

Periodic migrations are an integral part of the life history of many animals. Northern populations of Robins (*Erithacus rubecula*) migrate to the south [1], Equatorial Sandhoppers (*Talorchestia martensii*) migrate along a Y-axis perpendicular to the shoreline [2] and Alpine Newts (*Triturus alpestris*) migrate to their spawning pond [3]. All these and several other animal species are able to use magnetic cues for orientation [4,5]. Even though the phenomenon of magnetoreception in animals is well known, some fundamental issues, ranging from the underlying biophysical processes to the ability of navigation, are still poorly understood [6-8].

The most detailed studies in amphibian orientation biology were conducted with the Eastern Red Spotted Newt (*Notophthalmus viridescens*). These newts are able to orient themselves in the homeward direction after displacements of up to 45 km [9]. Magnetic orientation of the Eastern Red Spotted Newt is light-dependent [10,11] and experiments suggest that the inclination of the geomagnetic field is used in a magnetic map [9,12].

For anurans, only very limited information on magnetic orientation is available [13]. Tadpoles of the Bullfrog (*Lithobates catesbeianus*) and the Iberian Green Frog (*Pelophylax perezi*) could be trained to swim along a Y-axis, using the magnetic field as a cue [14,15]. In both species magnetic orientation by tadpoles is also light-dependent [16,17].

Adults of our target species, the European Common Toad (*Bufo bufo*), are able to locate their home pond even if it is filled up with soil [18]. Sinsch [19] provided the first evidence for a magnetic sense: he showed that toads were not able to initially orient towards their spawning pond after displacements over a few hundred meters when they had a bar magnet attached to their heads.

The use of arenas is a standard approach in orientation studies: Animals are placed in the centre of a circular test area and the point where they reach the perimeter is usually taken as measure of their orientation tendencies. Once orientation has been established, the magnetic field and other orientation cues can be manipulated and any changes in behaviour recorded [16,20,21]. This approach has been successfully applied to urodeles and larval anurans [10,16,22-24]. In experiments with amphibians, arena diameter usually ranged from 43 cm [25] to 80 cm [26]. Generally, arena experiments are more difficult to perform with adult anurans, because many species known for their impressive breeding migrations are large bodied, move faster than newts and salamanders and hence require larger arenas. Moreover, they tend to show escape behaviour that interferes with the realization of orientation trials.

We tested Common Toads in an arena situated outside of their home range, in order to investigate whether they would orient towards their breeding pond, and how their behaviour would be influenced by a change in direction of the magnetic field. To the best of our knowledge this study is the first experiment examining orientation of adult anurans with a controlled alignment and strength of the magnetic field.

## Methods

### Testing site

Experiments were carried out after sunset between 20:30 and 00:30 hours, between 28 March and 2 April 2010 in stable weather conditions (without rain or strong wind), at the car park of the Federal Research and Training Centre for Forests, Natural Hazards and Landscape (48°12'27" N, 16°13'45" E; 225 m a. s. l.), situated in Vienna, Austria.

### Experimental animals

**Figure 1:**
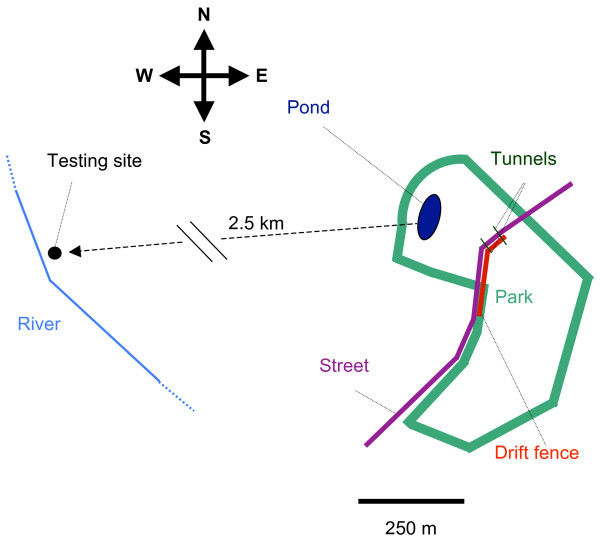
**Testing site**. Location of the pond in Vienna with the surrounding park and the direction towards the testing site. Toads are diverted by the drift fence until they can cross the street through the tunnels.

Only males of the Common Toad (*Bufo bufo*) were used for the experiments. This species is well known for its explosive breeding behaviour [27]. Many toads migrate after sunset during a period of approximately two weeks in spring. If streets intersect the migration route, traffic can cause high mortality. Therefore, barriers such as drift fences and amphibian tunnels are frequently built to reduce the death toll at roads. At our study site, a permanent drift fence constructed of wooden planks is positioned so as to direct the toads towards two tunnels along a mean direction of 32.5° (ranging from 5° to 60°), when north is set to zero (Figure 1).

The pond is located approximately 200 m from the fence and at a distance of about 2.5 km from the testing site. The general migration direction from the hibernation area to the pond was observed in the years 2009 and 2010 and estimated at approximately 318° (with a possible range of 285° to 351°), while the direction from the testing site to the pond was 85°.

We collected experimental animals at the drift fence during their migration to the spawning site (48°12'32" N, 16°15'47" E; 300 m a. s. l.) (Figure 1). During transport in a car to the testing site the toads were kept in closed buckets (filled with water to a level of 2 cm) to prevent access to chemical or visual cues. Animals were tested at the latest 3:49 hours after they arrived at the testing site.

### The arena

The toads were tested one by one in a circular arena (diameter 121 cm) consisting of a plastic wall (60 cm high) and a wooden floor. Wall and bottom were covered with black opaque plastic sheets. The toads could see the sky, whereas the horizon was not visible. Directions were painted on the wall of the arena with a white pen at five degrees intervals.

### Electromagnetic coils

**Figure 2:**
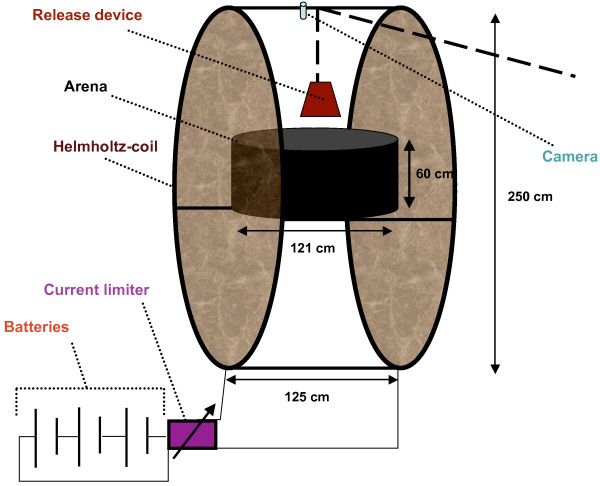
**Experimental set-up**. Experimental set-up with Helmholtz-coils. At the start of each trial the toad was placed in the centre of the arena and covered with a pot (release device).

Helmholtz-coils (21 windings per coil; approximately 50 A in the coils) were used to alter the alignment of the geomagnetic field [28] (Figure 2). When the coils were turned on the direction of magnetic north was rotated to 180°. The vertical field was not modified. Field strength was measured with a 3 D fluxgate magnetic field sensor (FLC3-70; Stefan Mayer Instruments). Three lead-acid batteries functioned as power supply; a current limiter and a resistor were used to hold the current constant at the required level.

### Testing procedure

At the beginning of each trial, a toad was placed in the centre of the arena and covered with the release device, a cylindrical opaque clay pot (diameter 20 cm). The toad was held under the release device to allow it to recover from handling. After 4 min the release device was lifted with rope and pulley, thereby releasing the toad into the arena. The behaviour of the toad (jumping and the directional choice at the wall) was recorded with observation positions being alternated after each trial to avoid bias. The point where a toad reached the wall for the first time was defined as the directional choice at this level. If a toad failed to reach the wall of the arena within 7 min, or if it was jumping rather than walking, it was excluded from further analysis. After every two trials alignment of the horizontal field was changed by 180°. Each toad was subjected to only one trial. Between trials the arena surface was wiped with paper towels to eliminate possible olfactory cues.

When the release device was lifted trials were recorded using an infrared camera (iSlim 321R; Genius). With these recordings we analysed the heading (alignment of the snout-vent axis) at the start of the trial (initial orientation) and the directional choice where the toad crossed a virtual inner circle (diameter 39 cm), which was electronically overlaid. The point at which the toad contacted the wall of the arena was recorded by the observer.

### Supplementary data

Data collected for each trial also included weather conditions (air temperature measured to the next 0.1°, cloudiness estimated to the next 5% and humidity measured to the next 0.1%), time of day, trial time (time a toad needed to reach the wall after the releasing device was lifted), and the toad's body length (measured to the nearest millimetre with a slide ruler). For the supplementary data standard deviations (SD) were calculated.

### Data analysis

Data were analysed using standard circular statistics [29,30]. Mean direction was calculated by vector addition and rounded to the nearest 5° (accuracy of observation). The Rayleigh-test was used to test for a non-random distribution. To test for bimodal orientation angles were doubled and multiples were reduced modulo 360° prior to analysis [29]. For each mean direction the mean vector length (*r*) was measured. The 95% confidence interval was determined for each significant mean vector. These analyses were carried out in Microsoft^® ^Excel^® ^2004 for Mac (Version 11.5.9). The statistical program R were used to plot the data [31].

To test for differences between bearings in the natural and the reversed magnetic field, the absolute deviations of the bearings of both distributions from the mean direction under the natural magnetic field (MDN) were calculated. Distributions of deviations were tested for departure from normality with the Kolmogorov-Smirnov-test. Then, one-sided *t*-tests were applied to examine whether absolute deviations from the MDN were significantly higher in the reversed field. Both tests were carried out using SPSS 13 for Mac OS X.

## Results

### Experimental animals

In total 62 toads with a mean body length of 67 mm (SD = 6 mm) were tested in the arena experiment. One toad jumped and three did not reach the wall after 7 min and were therefore excluded from analysis. Thus, in each set-up (natural and reversed magnetic field) 29 toads were tested successfully. Toads needed on average 3 min and 16 s (SD = 1 min and 31 s) to reach the wall after the release device was lifted (mean trial time).

### Experimental conditions

The strength of the geomagnetic field at the testing site was 40 μT, with an inclination of 63.4°. The declination for the first July in the testing year in Vienna was about 3°10' [32]. The strength of the reversed field differed by less than 2.9% from the geomagnetic field. The mean temperature was 7.8°C (SD = 3.3°C), the mean cloudiness was 45% (SD = 35%) and the mean humidity was 68.4% (SD = 5.6%). No successful trial was performed under complete cloud cover.

### Orientation in the natural magnetic field

**Figure 3:**
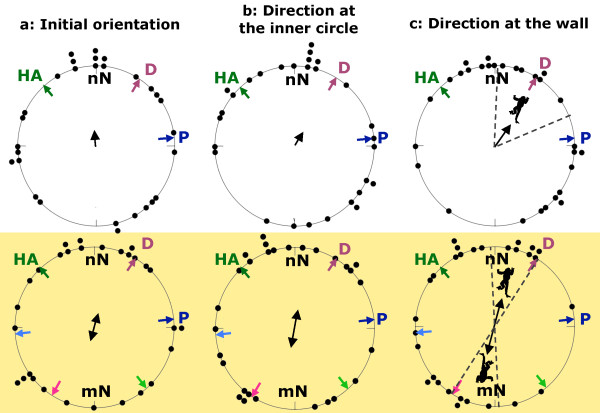
**Results**. Bearings and mean directions of the initial orientation (a), orientation at the inner circle (b) and orientation at the wall of the arena (c); in the figure the radius of the circle corresponds to a mean vector length (*r*) equal to one. Graphs in the upper row show the results obtained under natural conditions, graphs in the lower row (highlighted) those obtained with the reversed magnetic field (n = 29 in all cases). Dashed lines indicate the 95% confidence intervals in cases of significant orientation (*P *< 0.05). Under natural conditions the magnetic North (mN) coincided with the true north direction (nN), whereas in the reversed field mN was opposite to nN. Coloured arrows indicate the direction to the pond (P), the direction from the hibernation area to the pond (HA) and the mean direction along the drift fence (D). In the case of the reversed field, corresponding arrows are placed on the opposite side, in paler colours, to illustrate the directions when following only the magnetic field.

For initial orientation, no significant trend was detectable under the natural condition (Figure 3a, Table 1). Also at the inner circle a random distribution was observed (Figure 3b, Table 1). The distribution of bearings where toads contacted the arena wall was unimodal and the mean direction coincided with the mean of the former migration direction along the drift fence (d-axis, [33]) (Figure 3c, Table 1).

**Table 1:** Mean directions of toads in the natural and the reversed magnetic field

	Natural magnetic field			Reversed magnetic field			Comparison	
			
	Direction	*r*	*P*	Direction	*r*	*P*	*t*	*P*
Initial orientation	355°	0.20	0.33	15°/195°	0.18	0.41	-0.24	0.40
At the inner circle	25°	0.19	0.34	10°/190°	0.23	0.23	-0.78	0.21
At the wall	35°	0.34	0.03	15°/195°	0.35	0.03	-1.78	0.04

Mean directions of toads (n = 29 in all cases) were tested for significance using the Rayleigh-test; *r *represents the mean vector length; the absolute deviations from the mean vector under the natural field were compared between the distributions in the natural and in the reversed field using the *t*-test.

### Orientation in the reversed magnetic field

With the reversed magnetic field, initial orientations and the distribution at the inner circle were indistinguishable from random (Figure 3a, b, Table 1). In contrast, at the wall, the distribution of bearings was bimodally distributed, with the d-axis included in the 95% confidence interval (Figure 3c, Table 1), and failed to reach significance for a unimodal orientation (unimodal direction: 305°; *r *= 0.28; Rayleigh-test for unimodal distribution: *P *= 0.10, n = 29).

### Comparison of distributions

In no case distributions of deviations from the MDN differed significantly from normality (Kolmogorov-Smirnov-test: df = 29, *P* > 0.35). The differences between distributions at the start and at the inner circle were not significant (Table 1). At the wall in the reversed field, absolute deviations from the MDN were significantly higher than in the natural field (Table 1).

## Discussion

The arena we used was large enough for the Common Toads to manifest outcomes of orientation. Initial orientation, as well as the directions of movement at a short distance (39 cm) from the release point, were random, but a directional preference was evident when the toads reached the wall of the arena. A larger arena would perhaps allow to obtain clearer results; in displacement experiments with Common Toads in the field, Sinsch [19] included only individuals which had moved at least 5 m in the statistical analyses. With an increase in the size of the arena, however, manipulations of the magnetic field would become increasingly cumbersome.

The bearings at the wall of the arena were widely scattered, with the mean vector coinciding with the former migration direction along the drift fence. We tentatively interpret this result to show that toads oriented in their former migration direction. The variance of bearings may reflect at least partly their different prior migration directions up to or along the drift fence. It may appear implausible that toads should follow the course of the drift fence, rather than orienting in the direction from the hibernation area to the pond. The fence, however, had been installed for many years; thus, toads that migrate not for the first time may have learned the path to the tunnel and integrated it in an internal map.

Maintenance of the former migration direction has been found in some orientation studies with amphibians [33], whereas other experiments demonstrated the ability to orient to the breeding pond [9,12,13,26,34]. In the Common Toad, both outcomes have been obtained [19,35]; hence, these inconsistent results probably reflect differences in experimental design. Our approach differs in two ways from arena experiments that documented orientation towards the breeding pond: We collected the toads during their spawning migration, not at the breeding pond, and we allowed only a short recovery time between transport and orientation trials. Our rationale for taking toads during their migration was that we surmised migrating toads to be highly motivated to reach the pond, whereas toads already present at the pond might eventually lose the motivation to return due to exhaustion in their struggle for matings [36]. In preliminary trials at another testing site (900 m southeast from the spawning pond), toads that we had collected on the street exhibited orientation towards the breeding pond; reanalysis of these data showed, however, that this direction did not differ significantly from their likely former migration direction [37].

According to the magnetic map hypothesis [7,38], animals have to find their position on an internal map before they are able to choose the new direction towards their target. We speculate that, in our experiment, toads did not have sufficient time to assess their position in the interval between capture and testing, which ranged from a few minutes to 4 hours. With Common Toads displaced 3 km by Sinsch [19], it took up to three days before they headed homewards. In arena trials conducted by Buck [35] with Common Toads collected during their spawning migration, animals tested directly after displacement of 350 m maintained their migration direction, whereas a series with toads tested 24 hours after displacement resulted in orientation towards their breeding pond. Time dependence of orientation is also known in other vertebrates: Gray-Cheeked Thrushes (*Catharus minimus*), Swainson's Thrushes (*Catharus ustulatus*) and Greater Mouse-Eared Bats (*Myotis myotis*) need to recalibrate their magnetic compass with twilight cues for successful homeward orientation responses [39,40].

In the reversed magnetic field, bearings shifted from a unimodal to a bimodal distribution, along the same axis. This finding indicates an effect of the magnetic field on the orientation of the toads. Bimodality may result from a cue-conflict between the magnetic field and another cue. Individual preferences in using a multiple cue system have been reported in the orientation behaviour of anurans [41,42]. Hence, it is possible that some toads in our experiment followed magnetic cues in the reversed magnetic field, whereas others may have used celestial cues.

Because of its explosive breeding behaviour, high abundance, slow locomotion and high fidelity to its spawning ponds, the Common Toad is an excellent experimental animal in which to study orientation of anurans. The experimental set-up we developed opens possibilities to distinguish between multiple cues and to clarify the time-dependency of orientation systems. In future studies similar experiments with a longer resting time at the testing site should be conducted. Studies of magnetic orientation by juvenile toads, migrating away from their natal pond and by larvae trained to a Y-axis could lead to an integral picture of the life history of the Common Toad.

## Conclusion

We show that orientation of adult Common Toads can be investigated with a standard arena approach during their spawning migration. The distribution of directional choices changed when the horizontal component of the magnetic field was reversed. Bimodality in orientation under the altered magnetic field may have been caused by a cue conflict between magnetic and celestial cues.

## Competing interests

The authors declare that they have no competing interests.

## Authors' contributions

LL and GG conceived and organized this study, LL conducted the experiments and statistical analyses, LL and GG interpreted the results and wrote the paper. This work was submitted by LL to the Faculty of Life Sciences, University of Vienna, in partial fulfillment of the requirements for the degree of Mag. rer. nat.; GG was supervisor of this thesis. Both authors have read and approved the final manuscript.
